# Applying a high-throughput fluorescence polarization assay for the discovery of chemical probes blocking La:RNA interactions *in vitro* and in cells

**DOI:** 10.1371/journal.pone.0173246

**Published:** 2017-03-14

**Authors:** Gunhild Sommer, Alena Fedarovich, Venkatesh Kota, Reycel Rodriguez, Charles D. Smith, Tilman Heise

**Affiliations:** 1 Medical University of South Carolina, Department of Biochemistry & Molecular Biology, 173 Ashley Avenue, Charleston, SC, United States of America; 2 Department of Pharmaceutical and Biomedical Sciences, 173 Ashley Avenue, Charleston, SC, United States of America; Universitatsklinikum Carl Gustav Carus, GERMANY

## Abstract

The RNA-binding protein La is overexpressed in a number of tumor tissues and is thought to support tumorigenesis by binding to and facilitating the expression of mRNAs encoding tumor-promoting and anti-apoptotic factors. Hence, small molecules able to block the binding of La to specific RNAs could have a therapeutic impact by reducing the expression of tumor-promoting and anti-apoptotic factors. Toward this novel therapeutic strategy, we aimed to develop a high-throughput fluorescence polarization assay to screen small compound libraries for molecules blocking the binding of La to an RNA element derived from cyclin D1 mRNA. Herein, we make use of a robust fluorescence polarization assay and the validation of primary hits by electrophoretic mobility shift assays. We showed recently that La protects cells against cisplatin treatment by stimulating the protein synthesis of the anti-apoptotic factor Bcl2. Here, we show by RNA immunoprecipitation experiments that one small compound specifically impairs the association of La with Bcl2 mRNA in cells and sensitizes cells for cipslatin-induced cell death. In summary, we report the application of a high-throughput fluorescence polarization assay to identify small compounds that impair the binding of La to target RNAs *in vitro* and in cells.

## Introduction

In recent years, a growing number of RNA-binding proteins (RBPs) have been found to contribute to cancer development when aberrantly regulated at the expression level or misregulated by posttranslational modification[1–5]. Some of those RBPs belong to a family of RBPs referred to as La-related proteins (LARP)[6,7] and have been found to support tumor-promoting processes[1,8–12]. One member of the LARP family is the La autoantigen (La, LARP3), which is overexpressed in various types of tumor tissue and supports tumor pathobiology by promoting cell proliferation[13], motility and invasion[14], and anti-apoptotic processes[15]. The down regulation of murine La by RNA interference impairs tumor formation[16]. Previous studies suggest that the La protein facilitates the protein synthesis by binding to mRNAs encoding tumor-promoting and anti-apoptotic factors[13–17]. Hence, disrupting the interaction between RBP La and its target mRNAs might represent a novel approach in developing molecular drugs for anticancer treatment.

In addition to a role of La in tumor pathobiology, La supports viral replication by promoting viral protein synthesis or regulating viral RNA stability of life-threatening and incurable viruses such as hepatitis C virus (HCV), poliovirus, and hepatitis B virus (HBV)[18–21]. Although protein:RNA interactions play a critical role in tumorigenesis and viral infections, little is known about approaches targeting the interactions between cellular RNA-binding proteins and their target RNAs by small compounds[22–26].

**Fig 1:**
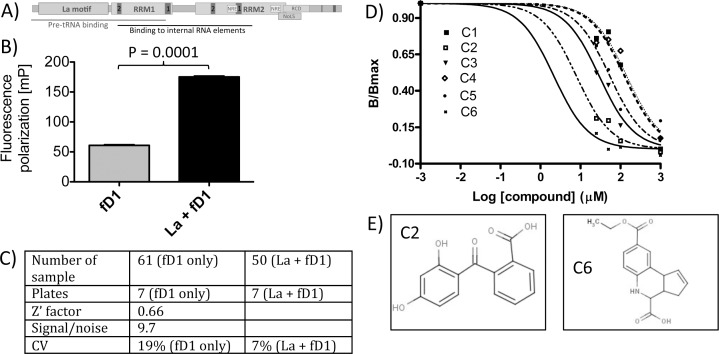
The La:RNA fluorescence polarization assay (La-FP assay). **A)** Domain structure of La wildtype (La-WT). RRM = RNA recognition motif with RNP1 and RNP2 consensus sequences. Lines indicate which domains are required for binding to different RNA substrates. NRE = nuclear retention element; NoLS = nucleolar localization signal; RCD = RNA chaperone domain; C-terminal dark bar = nuclear localization signal. **B)** Fluorescence polarization (FP) of the fluorescence-labeled cyclin D1-RNA oligonucleotide (fD1-RNA) in the absence and presence of recombinant La protein. **C)** Quality statistic for the La-FP assay. Z’ factor, signal to noise ratio and the coefficient of variation (CV) were determined by recording fluorescence polarization signals of fD1-RNA alone and in the presence of recombinant La. Measurements from seven microtiter plates performed at different days were combined to ensure proper statistics. **D)** La-FP on fD1-RNA in the presence of compound C1 to C6 at increasing concentrations. La protein (1.35 μM) was incubated with each compound at 25, 50, 100, 1000 μM for 30 min, followed by additional 15 min incubation with 0.8 μM fD1-RNA to detect the residual activity of the labeled fD1-RNA binding. Mean values of B/B_max_ (B_max_ = maximal binding in absence of compound) were plotted against the compound concentrations (μM). B_max_ = ΔmP_max_ = mPs−mP_free_ is the maximum specific binding, which defines complete saturation of La protein with fD1-RNA in the absence of a compound. B was determined as the difference between fluorescence polarization of fD1-RNA plus La plus compound and fluorescence polarization of fD1-RNA plus compound. IC_50_ values were determined using mean values of B/ B_max_ in a non-linear regression analysis by GraphPad Prism version 4.00 for Windows (GraphPad Software, Inc, San Diego, CA). **E)** The structures of the best two hits, C2 and C6, are shown.

The RNA-binding protein La binds to different classes of RNA molecules, such as pre-tRNAs, miRNA precursors, mRNAs, and viral RNAs[13,15,17,18,20,21,27–32]. However, a binding consensus motif has not been identified yet. The binding to RNA is mediated via three RNA-binding surfaces: the N-terminal La motif, and two RNA recognition motifs (RRM1 and RRM2)[6,33,34]. It is well established that the La protein binds to the 3’terminal poly(U) motif found in RNA polymerase III transcripts such as pre-tRNAs[35,36] and this binding is mediated by the concerted action of the La motif and RRM1[36]. Recent publications show that RRM1 and RRM2 are sufficient to bind internal RNA elements found in HCV, HBV, and cyclin D1 (CCND1) mRNA[13,37,38]. In addition, amino acids in the C-terminal domain of La might contribute to RNA binding[30,39,40]. These data show that the modular La protein binds different RNAs via different RNA binding surfaces and combinations of these surfaces (Fig 1A). Thus, targeted disruption of specific La:mRNA interactions could be used as a novel therapeutic strategy. It would be desirable to identify molecules that are able to block the binding of La to internal RNA elements in viral RNAs or mRNAs encoding tumor-promoting and anti-apoptotic factors, but which do not affect the binding of La to the e.g. 3’terminal poly(U) motif found in RNA polymerase III transcripts.

Recent progress has been made in targeting RBP:RNA interactions. Three small molecules isolated from microbial broth that inhibit the RNA-binding activity and functionality of the RNA-binding protein HuR have been identified[41]. More recently a high-throughput screening (HTS) assay for small molecules inhibiting HuR oligomerization and RNA binding has been completed.[42] Furthermore, a small compound was recently described that blocks the binding of the internal ribosome entry site (IRES) transacting factor hnRNPA1 to c-myc IRES and consequently reduces specifically c-myc IRES activity in cells at nM concentrations[43]. HCV IRES-mediated translation has been intensively studied, and molecules (benzoxazole scaffold) have been described that bind to the HCV IRES RNA and inhibit IRES-mediated translation at a concentration of 100 μM [44,45]. Efforts in targeting the RBP La led to the discovery of a La-derived peptide shown to impair HCV IRES-mediated translation[46] and a virtual screening identified a first La inhibitor able to impair the HBV life cycle at a concentration of 50 μM[47]. Moreover, eukaryotic initiation factors and their regulation by signaling pathways (e.g. AKT, mTOR) have been recognized as potential drug intervention points.[5,48,49]

Although initial steps have been taken to block viral infections with La inhibitors, the emerging role of La in tumor pathobiology prompted us to develop a novel screening technique that could identify heretofore unknown small compounds are able to inhibit La:RNA interactions.

Herein, we describe a robust high-throughput fluorescence polarization assay using a small, fluorescence-labeled RNA element–derived from CCND1 mRNA–as tracer and recombinant La protein as binding partner. We further describe the validation of the two best hits (C2 and C6). Additional 2D analogs (C2.01, C2.02, C6.01, C6.02) of those two compounds were tested for their activity to inhibit La:RNA interaction in electrophoretic mobility shift assays. All compounds were tested for cell toxicity on cancer cells and normal fibroblasts. By RNA immunoprecipitations, we demonstrate that C2.01 blocks the binding of La to specific mRNAs and sensitizes cells for cisplatin-induced cell death. Future studies should aim to optimize and develop those compounds to more potent and selective inhibitors for La:mRNA interactions in cell-based assays.

## Materials and methods

### Oligonucleotides

Please see supplemental information (S1 Table).

### Protein purification

For purification, the His-tagged recombinant human La wildtype (LaWT) and La-RRM1+2 proteins were expressed in *E*. *coli* BL21, then purified using Ni-NTA spin columns following the manufacturer’s instructions (Qiagen Ni-NTA Spin Handbook) as described in detail recently[30].

### Compounds

The DIVERSet library of 50,080 small compounds (10 mM in 100% DMSO) from ChemBridge Corporation (San Diego, CA) was provided by the MUSC Drug Discovery Core (DDC). The following identified compounds were purchased from Chembridge as well: C2.00 = product number 5629492 (MW 258), C2.01 = product number 5626971 (MW 256, 2D stereotype of C2.00 based on structural similarity), C2.02 = product number 5475540 (MW 292, 2D stereotype of C2.00 based on structural similarity), C6.00 = product number 5667569 (MW 287), C6.02 = product number 6964444 (MW 315, 2D stereotype of C6.00 based on structural similarity), C6.02 = product number 6994592 (MW 315, 2D stereotype of C6.00 based on structural similarity). The compounds were solubilized at a concentration of 10 mM in 100% DMSO and stored at -80°C.

### Fluorescence polarization high-throughput screening (HTS) assay

Measurements of fluorescence and fluorescence polarization were performed on a Spectramax M5 microplate reader (Molecular Devices, Sunnyvale, CA) with the excitation and emission wavelengths 544 nm and 590 nm, respectively. To block unwanted residual excitation light and minimize background interference, a 570 nm “cutoff” filter was applied. Black shallow 384-well micro plates (ProxiPlate ^TM^– 384 F Plus, PerkinElmer) were used to record data. To minimize the polarization effects from fluorophore, which may be bound to the surface of the well, both excitation and emission data were recorded from the top of the well. Reading time was 100 ms per well. Millipolarization units (mP) were used to express fluorescence polarization values defined by the equation mP = 1000 x [(I_v_—GI_h_) / (I_v_+ GI_h_)], where I_v_ and I_h_ are parallel and perpendicular emission intensity measurements corrected for background (buffer), and G-factor.

The ratio of sensitivities of the detection system for vertically and horizontally polarized light G = I_v_/I_h_ was determined with dilution series of the fluorescence-labeled cyclin D1-RNA oligonucleotide (fD1) only (free-tracer) using SoftMax® Pro Data Acquisition & Analysis Software (Molecular Devices, Sunnyvale, CA). The assay window–the specific binding ΔmP–was defined as the difference between fluorescence polarization (mP) for La protein:fD1 reactions (mP_s_) and fluorescence polarization of free-fD1 reactions (mP_free_): ΔmP = mPs−mP_free_.

The assay was formatted using 10 μl reaction volume per well. Assay buffer (20 mM Tris-HCl pH 7.5, 3.0 mM MgCl_2_, 0.5 mM EDTA, 150 mM NaCl, 0.05% IGEPAL, BGG 100 μg/ml (BGG = Bovine γ-Globulin or γ-Globulins from bovine blood)) was used to dilute all ingredients. Detergent was added to prevent the potential aggregation of small compounds. The aggregation of small compounds can lead to false-positive results in HTS approaches and is indicated by a high Hill factor[50]. To find the optimal concentration of the free fluorescent tracer (fD1), which gives low and stable fluorescence polarization signal, and to calculate the G-factor, 100–1000 nM fD1 dilution series were measured in quadruplicates. Using variable fD1 concentrations, the optimal tracer:protein ratio was determined in binding experiments with increasing concentrations of La protein (1.35–1350 nM). To start the reaction, fD1 and La protein stock solutions were mixed into each well. Each reaction was performed in quadruplicates. After shaking the plate for 2 min followed by 15 min incubation at room temperature, fluorescence polarization signals were recorded as described above. Fluorescence polarization measurements were performed on different days using 7 microtiter plates (111 samples total).

Assay performance was assessed using the following parameters: the signal-to-noise ratio = (μ_Ps_ - μ_Pfree_)/SD_Pfree_ and the Z’ factor. The latter was calculated as Z’ = 1- (3SD_Ps_ + 3SD_Pfree_)/(μ_Ps_-μ_Pfree_), where SD_Ps_ and SD_Pfree_ are standard deviations, and μ_pc_ and μ_Pfree_ are means of recorded polarization values of P_s_ (fluorescence polarization of La protein mixed with fD1) and P_free_ (fluorescence polarization of fD1 only), respectively. The coefficient of variation (CV) is given in percent and reflects the ratio between the standard deviation and the mean[51].

High-throughput screening against the ChemBridge DIVERSet library was carried out under the following conditions: 1 μl of each compound (dissolved in 100% DMSO) was pre-incubated with 8 μl of La protein for 30 min at room temperature, followed by an additional 15 min incubation with 1 μl of fD1 (final concentration of La, fD1, and DMSO in the 10 μl reaction: 1.35 μM La, 0.8 μM fD1, 10% DMSO). The reactions were performed in duplicate. In addition to samples with the compounds, each plate contained 2 background wells (10 μl buffer only), and at least 4 wells for P_s_ (La and fD1) and P_free_ (fD1 alone) reactions. All samples included 10% DMSO. Pilot screening was carried out with 10 mM cocktails of 10 compounds (giving a final concentration of 100 μM for each compound). To evaluate potential inhibitory activity of individual compounds present in compound cocktails that showed 80% or more reduction in ΔmP compared to maximum binding (ΔmP_max_ = mPs−mP_free_), each compound was then tested individually in concentration-response experiments. Using the same fluorescence polarization assay format, La protein (1.35 μM) was incubated with each potential hit compound at 25, 50, 100, 1000 μM (in triplicate) for 30 min, followed by an additional 15 min incubation with 0.8 μM fD1. Data points for specific binding (B) were normalized to the maximum specific binding (B_max_), which defines complete saturation of the La protein with fD1 in the absence of a compound (B/Bmax). IC_50_ values were determined through non-linear regression analysis using GraphPad Prism version 4.00 for Windows (GraphPad Software, Inc., San Diego, CA).

### Electrophoretic mobility shift assay (EMSA)

12% native TBE polyacrylamide gels were prepared in a Mini-PROTEAN Tetra Handcast System (BioRad). The gel was pre-run for 30 min at 140 V in 1x TBE buffer (45 mM Tris/HCl pH 8.5, 45 mM boric acid, 1 mM EDTA). To prepare the RNA-mix, fluorescence labeled RNA oligoribonucleotides (oligos) were diluted in fluorescence polarization buffer (20 mM Tris/HCl (pH 7.4), 150 mM NaCl, 3.0 mM MgCl_2_, 0.5 mM EDTA, 0.05% (v/v) IGEPAL), mixed for 10 min at 80°C, and then immediately placed on ice for at least 5 min. All RNA oligos were used at a final concentration of 50 nM. Recombinant La protein was diluted in D-buffer (20 mM Tris/ HCl (pH 7.4), 150mM NaCl, 3mM MgCl_2_, 0.5mM EDTA, 5% glycerol) and placed on ice until use.

La titration was performed at final concentrations of 20, 40, 60, 100, 200, 300, 400, 500, 600 nM La. Three different La concentrations were used for compound studies: 60 nM for fD1 RNA, 200 nM for fPolyU RNA, and 150 nM for TOPf RNA studies. Two μl of diluted compound (diluted in 100% DMSO) or 2 μl 100% DMSO (control reaction) was combined with 8 μl La-mix in a 96-well plate. 10 μl RNA-mix was added, mixed for 2 min, and incubated for 10 min at RT in the dark. Final concentrations in the reaction: 18 mM Tris/HCl (pH 7.4), 135 mM NaCl, 2.7 mM MgCl_2_, 0.45 mM EDTA, 0.025% (v/v) IGEPAL, 2% glycerol, 10% DMSO). Samples were loaded onto the pre-run gel without any loading buffer. Gels were run at 160 V for 40 min in the dark. The gels were carefully transferred to a clear plastic wrap and scanned using a Typhoon FLA900 imager. The La-RNA complex (La-RNP) formation was quantified using the ImageQuant TL software.

### Cell culture

Normal fibroblast MRC5 and human embryonic kidney HEK293 cells were purchased from ATCC. Head and neck squamous cell carcinoma cell lines UM-SCC 22A (SCC 22A) and UM-SCC 22B (SCC 22B) were described elsewhere[14,15,52]. MRC5 cells were cultured in EMEM plus 10% FBS; SCC 22A, SCC 22B, and HEK293 cells were cultured in advanced DMEM (Gibco) containing 2 mM L-glutamine (Life Technology) and 10% FBS. All cell lines were tested for mycoplasma contamination by applying a MycoSensor PCR Assay kit according to the manufacturer’s instructions (Agilent Technologies).

To test for cell toxicity the compounds were dissolved in 100% DMSO to a concentration of 100 mM. Subsequently, compounds were diluted in media to 10 mM (10% DMSO), 1 mM (1% DMSO) and tested at 150, 125, 100, 75, 50, 25 μM in SCC 22B and MRC5 cells. To reach this concentration compounds (stock 1 mM, 1% DMSO) were diluted in the cell culture dish (96-well format, 100 μl final volume) as follows: 150 μM = 6.7 fold (final DMSO: 0.15%); 125 μM = 8.0 fold (final DMSO: 0.125%); 100 μM = 10 fold (final DMSO: 0.1%); 75 μM = 13.3 fold (final DMSO: 0.075%); 50 μM = 20 fold (final DMSO: 0.05%); and 25 μM = 40 fold (final DMSO: 0.025%). As a control the highest DMSO concentration of 0.15% was used. Cells were cultured in appropriate media (see above) for 48h. Subsequently, cells were washed twice with 1x PBS and quantified after staining with fluorescence dye (*CyQUANT®*, Life Technologies). For this experiments 3x10^4^ of MRC5 and 1x10^4^ SCC 22B cells were plate the day before the experiment.

To test whether compound C2.01 sensitizes cells for cisplatin, cells were treated with 0.2% DMSO alone, cisplatin at 4, 8, 16, 32 or 64 μM concentration alone or in combination with 50 μM compound C2.01. The half maximal inhibitory concentration (IC_50_) of cisplatin was determined by treating SCC 22B cells with increasing cisplatin (Selleckchem) concentrations or vehicle (DMSO) for 48 h (96-well format) as described recently[15]. For this experiments 1x10^4^ SCC 22B cells were plated the day before the experiment. DMSO or C2.01 was added to a final concentration of DMSO (0.05%) and C2.01 (50 μM). Six hours later the different cisplatin concentrations were added. The highest DMSO concentration of 0.25% was used in the control for cisplatin and C2.01 double treatment. Subsequently, cells were washed twice with 1x PBS and quantified after staining with fluorescence dye (*CyQUANT®*, Life Technologies) 48 after the initial C2.01 treatment.

### RNA immunoprecipitation (RIP)

RIP experiments were performed with HEK293 stably expressing gfp (green fluorescent protein)-tagged La wiltype (La-WT) protein as described recently[29]. The compound C2:01 was dissolved in 100% DMSO to a concentration of 100 mM. Subsequently, the compound was diluted in media to 10 mM (10% DMSO) and 1 mM (1% DMSO). The 1 mM stock was diluted 6.7-fold in culture media to reach a final concentration of 150 μM (0.15% DMSO). Cells were washed with ice-cold 1x PBS, and lysed by incubating with lysis buffer (20 mM Tris-HCl, (pH 7.4), 150 mM NaCl, 1% IGEPAL CA-630, 10% glycerol, 1 mM EDTA, 50mM NaF, and 1 mM DTT, supplemented with RNase inhibitors and protease inhibitors) on tube rotator for 15 min at 4°C. The cell lysate was sonicated (3 sec for 10 times at power 3, Sonic Dismembrator Model 100) and cleared by centrifugation at 14,000 g at 4°C for 20 min. The cleared lysate was incubated with anti-gfp magnetic beads (MBL International) on the orbital rotor at 4°C overnight. The beads were washed four times with wash buffer I (50 mM Tris-HCl (pH 7.4), 300 mM NaCl, 0.05% IGEPAL CA-630, 20 mM EDTA, 1 mM DTT, and 1 mM MgCl_2_) and three times with wash buffer II (50 mM Tris-HCl (pH 7.4), 300 mM NaCl, 0.05% IGEPAL CA-630, 20 mM EDTA, 1 mM DTT, 1 mM MgCl_2_ and 1 M urea)[29].

For preparation of the RNA, the beads were resuspended in wash buffer II and RNA was extracted by applying the Phenol-Chloroform-Isoamyl alcohol method. Phenol-Chloroform-Isoamyl alcohol (25:24:1, v/v, Sigma-Aldrich) was added, the sample was vortex and heated for 10 min at 65°C. After centrifugation for 20 min at 17,000 g at room temperature the aqueous phase was transferred to a fresh tube and 1μl GlycoBlue (Ambion) and 600 μl Isopropanol was added. The sample was vortexed and precipitated over night at -20°C, centrifuged for 20 min at full speed and 4°C, and the RNA pellet was washed once with 800 μl cold 70% ethanol. The RNA pellet was dissolved in RNase-DNase-free H_2_O and quantitated by NanoDrop spectrophotometer and subjected to RT-qPCR analysis. By RT-qPCR the La-specific RIP-RNA pellets (gfp-tagged La) were tested for the presence of Bcl2, CCND1, L37, L22 and S6 mRNA and none of those were detected in the control RIP experiments (gfp alone). The enrichment was calculated by establishing standard curves for specific mRNAs using the RT^2^ qPCR Primer Assays (Qiagen): Bcl2 (PPH00079B), CCND1 (PPH00128F), L37 (QT00014105), L22 (QT00079982), S6 (QT02505328) as described recently[29]. The RNA binding between DMSO control and C2.01-treated was compared using the formula: [treated (RIP/input) / DMSO control (RIP/input)] X 100. Immunoblotting was performed using anti-gfp antibodies (Roche) to assess pull-down efficiency of gfp-tagged La and gfp control in the RIP assay.

## Results

### Applying of a high-throughput La:RNA fluorescence polarization assay

It has been shown previously that the La protein supports tumor-promoting and anti-apoptotic cellular processes[13–17,31]. The mapping of the La binding site in CCND1 mRNA[30] allowed us to develop an La:RNA fluorescence polarization (La-FP) assay to screen for small biologically active molecules that inhibit the binding of La to the CCND1 mRNA derived RNA element, referred to as fD1[30]. The principle of an fluorescence polarization assay relies on a “small” fluorescence-labeled tracer and a “large” binding partner. In solution, the tracer tumbles and unpolarized light is emitted after excitation. However, when bound by a binding partner, the tumbling is reduced and more polarized light can be measured after excitation. In our La-FP assay, we used the fluorescence labeled fD1 RNA oligo as a tracer and first measured the fluorescence of the tracer alone. At a concentration of 100–200 nM the recorded average fluorescent signal of the free fD1 RNA oligo was low (85–108 relative units (RU)) but reached 339–583 RU at 400–1000 nM fD1 (data not shown). This strong signal is important because the fluorescence signal should be intense enough to overcome auto-fluorescence of potential fluorescent compounds present in the library.

Next we determined the fluorescence polarization of free fD1 RNA oligo at 100–200 nM (148 mP) and 400–1000 nM (110 mP) concentrations (data not shown). Finally, we determined the fluorescence polarization of La:fD1 reactions at various protein:RNA ratios to define the optimal assay window. With the increase of the La protein concentration, the greater amount of fD1 RNA oligos was bound to the protein, and fluorescence polarization increased to the maximum of 150–234 mP at different RNA concentrations (not shown). The best assay window of 107 mP was determined at a La:fD1 ratio of 1.7 (1350 nM La: 800 nM fD1) (Fig 1B).

To characterize the La-FP assay in depth, we determined two critical parameters for quality statistics (Fig 1C)[51,53]. One important criterion reflecting the suitability of an high-throughput assay is the Z-factor, which can be calculated from a number of repeated reactions to determine whether the response is large enough to obtain reliable data. The Z’ factor considers mean signals of the sample and of the control as well as their standard deviations. An optimal assay has a theoretical maximal dimensionless Z’ value of 1. Assays with a Z’ factor above 0.6 are considered as robust. A Z’ factor value of 0.66 was calculated from independent fluorescence polarization measurements performed on different days using 7 microtiter plates (111 samples total). Another important criterion is the coefficient of variation (CV), which describes the accuracy and repeatability of an assay as a percentage. The CV reflects the ratio between the standard deviation and the mean. No variation would result in a CV = 0%. We calculated a CV for the control (fD1 oligo alone) of 19% and a CV for La:fD1 samples of 7%, reflecting a low variation.

Library compounds are often solubilized in DMSO, and therefore we tested various DMSO concentration and found that 10% DMSO is not affecting the La-FP assay outcome (data not shown), which would reflect the maximal DMSO concentration of a 1:10 diluted compound in our assay (maximal concentration of compound tested = 1 mM, compound stock concentration = 10 mM).

In short, a Z’ factor above 0.6 and a low CV demonstrates that the La:fD1 fluorescence polarization assay is robust and reproducible.

### Pilot screen using the La-FP assay

After successful adaptation of the La-FP assay to the 384-well plate format, we used the DiverSet ChemBridge small compound library for a pilot screen. We screened a total of 1152 compound pools with each pool containing 10 compounds (12 combination plates: 10 compounds/well = 960 compounds/plate, total number of compounds tested 11,520). The final concentrations in the reaction were: 100 μM of each of the 10 compounds, 1.35 μM of recombinant La protein, and 0.8 μM of fD1-RNA oligo in a 10 μl reaction volume. Seven active pools were identified. To evaluate the inhibitory activity of the individual compounds found through the fluorescence polarization screening, we used 4 concentrations (3 repeats each). Each potential hit compound at 25, 50, 100, 1000 μM was incubated with La protein followed by the addition of the fluorescent RNA fD1. Data points were normalized to the maximum specific binding ((ΔmP_max_ = mPs−mP_free_), which defines complete saturation of the La protein by fD1. Our analysis led to the identification of six compounds inhibiting La:fD1-RNA complex formation referred as hits (initial hit rate: 0.05%) with IC_50_ values between 50 and 2.5 μM (Fig 1D). Since compounds C6 and C2 had 100% La-fD1 inhibition at 100 μM and 1000 μM and the mP of the free label-compound (mP_free_) was slightly lower than that of a free RNA label alone is explaining why the data points at 100 μM and 1000 μM are below 0 (Fig 1D). We applied a cut-off at IC_50_ values >10 μM as a criteria for the best hits. As a result two compounds C6 (8-(ethoxycarbonyl)-3a,4,5,9b-tetrahydro-3Hcyclopenta[c]quinoline-4-carboxylic acid) and C2 (2-(2,4-dihydroxybenzoyl)benzoic acid) (Fig 1E) were chosen for orthogonal testing using electrophoretic mobility shift assays (EMSAs).

### Binding of La to different RNA molecules

**Fig 2:**
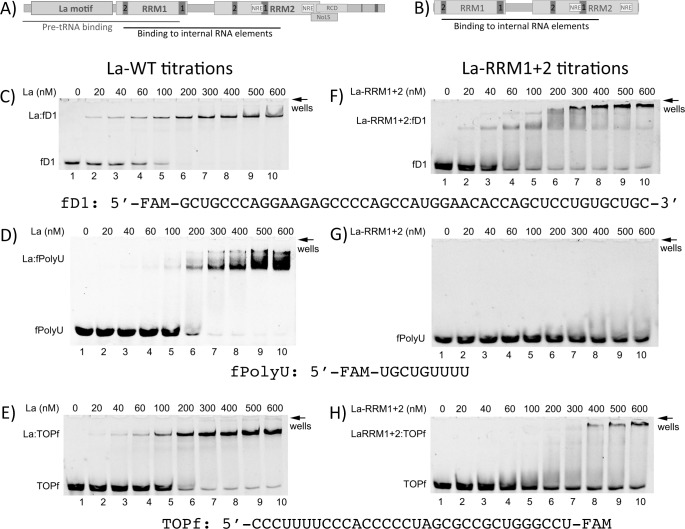
Binding of La wilstype and the minimal RNA-binding competent La-RRM1+2 mutant to different RNA substrates. Domain structure of La wildtype (La-WT) **(A)** and La-RRM1+2 mutant **(B).** Either recombinant La-WT or La-RRM1+2 protein was titrated into binding reactions containing 50 nM fluorescence labeled fD1-RNA **(C, F)**, fPolyU-RNA **(D, G)**, or TOPf-RNA oligoribonucleotides **(E, H)**. The EMSA reactions were loaded without dye and separated on 10% native polyacrylamide gels. Representative gels of independent experiments are shown. All experiments were performed at least three times.

Before testing the hits in EMSAs, we tested binding of recombinant La wildtype (La-WT) and the minimal RNA-binding competent La-RRM1+2 to different RNA substrates. The RBP La binds to different RNA molecules–such as pre-tRNAs, miRNA precursors, and mRNAs encoding cellular and viral factors–by using three main RNA-binding motifs: the La motif (LAM), RRM1 and RRM2(Fig 2A). We have shown in other studies that La binds to HBV RNA, CCND1, and Bcl2 mRNA in cells and mapped small binding sites in those RNAs *in vitro*[13,15,37]. We found that a La fragment, referred as La-RRM1+2, containing RRM1 and RRM2 is sufficient for binding to those internal RNA elements (Fig 2B)[30,37]. Before we validated the hits we identified in the La-FP assay (Fig 1D and 1E), we first compared the binding of La-WT (Fig 2C, 2D and 2E) and La-RRM1+2 (Fig 2F, 2G and 2H) to different known La target RNAs: i) 5’ fluorescence-labeled structured RNA element representing a La binding site in CCND1 mRNA (fD1[30], Fig 2C and 2F), ii) 5’ fluorescence-labeled polyuridylated RNA oligo (fPolyU, Fig 2D and 2G) previously used to study the binding of La to the 3’-terminal end of RNA polymerase III transcripts and to crystalize a La:polyU complex[36], and iii) 3’ fluorescence-labeled 5’-terminal oligopyrimidin (TOPf) tract element (TOPf, Fig 2E and 2H) derived from the mRNA encoding the large ribosomal protein RPL5. TOP mRNAs are bound by La *in vitro* as well as in cells[54,55]. EMSAs were used to determine the binding of the fluorescence-labeled RNA oligonucleotides (oligo) to increasing La concentrations. The predicted secondary structures of all RNA molecules used are presented (Panels A, B, C in S1 Fig).

As recently shown, the fD1-RNA is efficiently bound by La-WT protein, forming a single La:fD1 complex with a K_D_ of 85 +/- 7.5 nM (n = 2) which is similar to the K_D_ of 80.3 nM published recently (Fig 2C [30]). The fD1 oligo is also efficiently bound with a K_D_ of 81.7 +/- 12.6 nM (n = 3) by recombinant La-RRM1+2, however, multimeric complexes appear at higher protein concentrations, suggesting La-RRM1+2:D1f aggregation, probably formed by RRM mediated protein interactions (Fig 2F). The fPolyU oligo is bound by La-WT with a K_D_ of 138.3 +/- 12.6 nM (n = 3) and the formation of multimeric complexes (Fig 2D). Since the La motif is required for binding fPolyU, it was expected that La-RRM1+2 would not bind the fPolyU oligo (Fig 2G). Binding of La to TOP elements by recombinant La has been reported in the past[55] and was confirmed by demonstrating that La-WT binds the TOPf oligo with a K_D_ of 146.7 +/- 35.1 nM (n = 3) (Fig 2E). However, the binding of the TOPf oligo by La-RRM1+2 is very inefficient, and very high concentrations (>300 nM,) of La-RRM1+2 are required to shift a portion of the TOPf oligo (Fig 2H, no reliable K_D_ was calculated), suggesting that other domains of La are required for robust binding of TOP elements. In addition, multimeric La-RRM1+2:TOPf (Fig 2H) complexes are formed similar to the La-RRM1+2:D1f interaction (Fig 2F).

**Fig 3:**
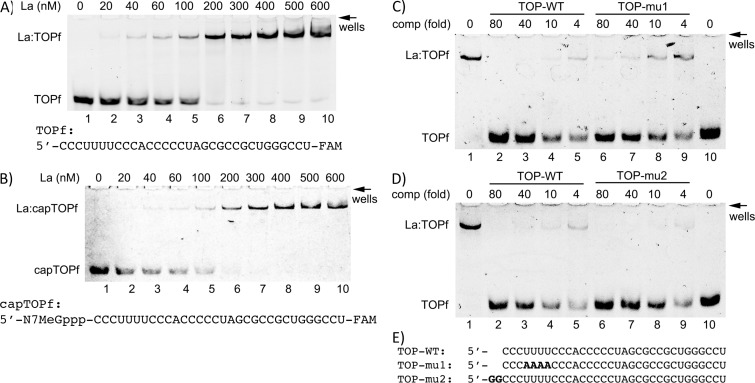
Characterization of La binding to the TOP element of RPL5 mRNA. **A)** and **B)** Comparison of La binding to TOPf- or 5’ capped TOPf-RNA (capTOPf) oligonucleotide (50 nM). Recombinant La-WT was titrated into binding reactions contain fluorescence labeled TOPf-RNA **(A)**, or fluorescence labeled capTOPf-RNA **(B)** oligoribonucleotides (both at 50 nM) and analyzed on a 10% native polyacrylamide gel. Representative gels of independent experiments are shown. **C)** and **D)** Competition experiments using three different RNA oligonucleotides **(E)**. Recombinant La-WT (100 nM) was used in binding reactions contain TOPf-RNA (50 nM) and increasing concentrations of unlabeled competitor RNA oligonucleotides: TOP-WT, TOP-mu1 or TOP-mu2. Reactions were analyzed on a 10% native polyacrylamide gel. **E)** Sequence of unlabeled competitor RNA oligonucleotides used in this study. All experiments were at least performed twice.

To our knowledge, the influence of a cap structure (7-methyl-guanosin (m7GpppN)) at the 5’-end of TOP mRNAs on La binding has not been investigated. To test whether the binding of TOPf by La is affected by a, cap structure, we used a synthetic 5’-end capped TOPf oligoribonucleotide (capTOPf) and demonstrated that the cap structure is not interfering with the binding of La (Fig 3, compare A and B, capTOPf:LaWT, K_D_ = 183.3+/- 15.3 nM). Since the cap is blocking the 5’end, this finding suggests that La is not necessarily recognizing the immediate 5’-end of the oligo but rather binds uridyl stretches within the TOPf oligo. To test whether a 5’-terminal pyrimidine or an internal stretch of uridine nucleotides is important for La recognition, we performed competitive EMSAs using TOPf oligos and unlabeled mutants (Fig 3E): TOP-mu1 (change of internal uridines to adenosines) and TOP-mu2 (two purines at the 5’-end of the oligo). Competitive EMSAs suggest that TOP-WT and TOP-mu2 efficiently compete for binding, suggesting that purines at the 5’end are not impairing binding (Fig 3D). However, TOP-mu1, with uridine to adenosine changes in the internal pyrimidine stretch, was a weak competitor (Fig 3C). This finding suggests that La recognizes the internal stretch of uridines in the TOP element of rpL5.

In sum, La-WT binds the fD1- and TOPf-RNA oligos at a low nM concentration range (20–40 nM), whereas fPolyU RNA oligos are bound at higher nM concentrations (>100 nM). La-RRM1+2 binds the D1f oligo with high affinity, whereas TOPf is only weakly bound, and fPolyU not at all. Therefore, we conclude that the RRM1 together with RRM2 is sufficient for binding structured RNA oligos (compare Panels A, B, C in S1 Fig). Small PolyU RNA and less structured RNA oligos (TOP) require additional binding surfaces to establish a robust complex, suggesting that different binding modes of La are involved in interacting with these three classes of La target RNAs.

### Validation of compound C6 and C2 in orthogonal tests

Next we used our established EMSA to test whether compound C2 and C6 can compete against the three RNA oligos: fD1, fPolyU, and TOPf. All of the EMSA reactions were loaded in the following order: lane 1 contains the RNA oligo alone; lane 2 contains the RNA oligo plus La protein at concentration that shifts 50% of the RNA oligo (positive control); lanes 3–9 contain the RNA oligo, La protein and decreasing concentrations of the compound; and lane 10 contains the RNA oligo and the highest compound concentration (negative control).

**Fig 4:**
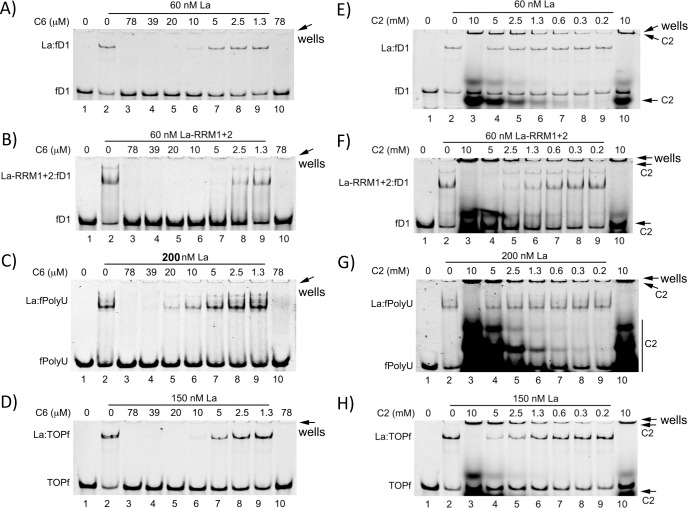
Compound C6 and C2 impair La binding to different RNA substrates. Constant concentration of recombinant La as indicated and fluorescence labeled RNA oligonucleotides (50 nM) were used with increasing concentrations of compound C6 or C2, respectively. Lane 1 and 2 contained the highest vehicle (DMSO) concentration used with the compounds. The compound was first incubated with La and RNA oligoribonucleotides were quickly added thereafter. Lane 10 always contained the fluorescence labeled RNA oligoribonucleotides and the highest concentration of compound tested. Reactions were analyzed on a 10% native polyacrylamide gel and representative gels of independent experiments are shown. **A)** fD1-RNA, La-WT (60 nM) and decreasing concentrations of C6, **B)** fD1-RNA, La-RRM1+2 (60 nM) and decreasing concentrations of C6, **C)** fPolyU-RNA, La-WT (200 nM) and decreasing concentrations of C6, **D)** TOPf-RNA, La-WT (150 nM) and decreasing concentrations of C6, **E)** fD1-RNA, La-WT (60 nM) and decreasing concentrations of C2, **F)** fD1-RNA, La-RRM1+2 (60 nM) and decreasing concentrations of C2., **G)** fPolyU-RNA, La-WT (200 nM) and decreasing concentrations of C2, **H)** TOPf-RNA, La-WT (200 nM) and decreasing concentrations of C2. All fluorescence labeled RNA oligoribonucleotides were used at a concentration of 50 nM. All experiments were performed at least twice.

By comparing compound C2 and C6 in EMSAs, we found that C2 but not C6 displays auto-fluorescence as indicated by the arrows (Fig 4, compare A and E). The auto-fluorescence signal of compound C2 was visible in the lower part of the native gel as well as in the pockets (Fig 4E–4H, lane 10).

As shown, both C2 and C6 were able to prevent La:fD1 complex formation (Fig 4A and 4E). However, C6 was a more potent compound inhibiting La:fD1 complex formation at 10 μM. In contrast, C2 was impairing La:fD1 complex formation only at the highest concentration (10 mM) tested. The auto-fluorescence of C2 is probably the reason for the overestimated IC_50_ for C2 in La-FP assay (Fig 1D). These data show the strength of the EMSA in visualizing La:RNA complexes and potential auto-fluorescence of compounds.

Next, we tested whether C2 and C6 were also impairing the binding of fD1 to La-RRM1+2. We found that C6 as well as C2 blocked La-RRM1+2:fD1 complex formation at 10 μM or 5 mM, respectively (Fig 4, compare B and F).

To evaluate whether the compounds selectively impair La:RNA complexes, we tested whether the compound C6 and C2 are inhibiting the binding of the fPolyU oligos, recently described to be bound mainly via the La motif and the RRM1[36] and shown above (Fig 2). Our data show that C6 blocked La:fPolyU complex formation at 40–20 μM C6 (Fig 4C) and therefore at slightly higher concentration than the La:fD1 complex formation (Fig 4A). As shown for La:fD1 interaction (Fig 4E), compound C2 impaired La:PolyU complex formation only at the highest concentration tested (10 mM, Fig 4G). Finally, we tested whether C2 and C6 can compete for La:TOPf complex formation. Titration of C6 into the La-WT:TOPf reaction blocked complex formation at 10 μM concentration (Fig 4D) as observed for the La:fD1 complex (Fig 4A). Again, C2 impaired complex formation only at the highest concentration tested (Fig 4H).

The data suggest that interaction between the La protein and internally structured RNA, as present in the D1f-RNA oligo, and terminal oligopyrimidine stretches, as present in the TOPf oligo, can be efficiently blocked *in vitro* by compound C6. In contrast, C6 was a weaker competitor for the La:fPolyU complex formation, suggesting that some specificity of C6 for La:fD1 and La:TOPf complexes exists *in vitro*.

### 2D analogs of C2.00 and C6.00 inhibit La:fD1 interactions

**Fig 5:**
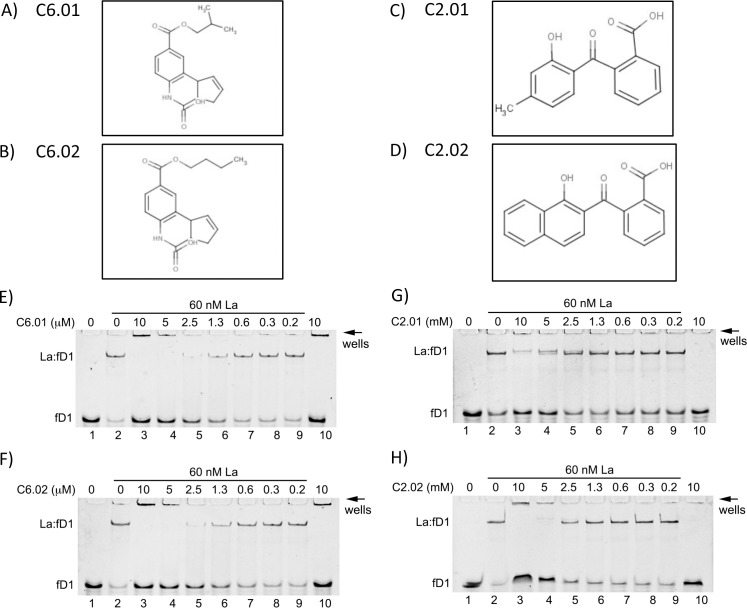
The 2D analogs C6.01, C6.02 and C2.01, C2.02 compete for La binding to fD1-RNA. Chemical structure of 2D analogs of C6 and C2, respectively, are shown: C6.01 **(A)**, C6.02 **(B)**, and C2.01 **(C)**, C2.02 **(D)**. Constant concentration of recombinant La protein (60 nM) and fluorescence labeled RNA oligonucleotides (50 nM) was used with increasing concentrations of compound C6.01, C6.02, C2.01 or C2.02. Lanes 1 and 2 contained the highest vehicle (DMSO) concentration used with the compounds. The compound was first incubated with La and RNA oligonucleotides were quickly added thereafter. Lane 10 contains the fluorescence labeled RNA oligonucleotides and the highest concentration of compound tested. Reactions were analyzed on a 10% native polyacrylamide gel and representative gels of independent experiments are shown. **E)** fD1-RNA, La-WT and decreasing concentrations of C6.01, **F)** fD1-RNA, La-WT and decreasing concentrations of C6.02, **G)** fD1-RNA, La-WT and decreasing concentrations of C2.01, and **H)** fD1-RNA, La-WT and decreasing concentrations of C2.02. All experiments were performed at least twice.

We investigated two commercially available 2D analogs of C6 (C6.01 (8-(isobutoxycarbonyl)-3a,4,5,9b-tetrahydro-3H-cyclopenta[c]quinoline-4-carboxylic acid) and C6.02 (8-(butoxycarbonyl)-3a,4,5,9b-tetrahydro-3H-cyclopenta[c]quinoline-4-carboxylic acid), Fig 5A and 5B) and two commercially available 2D analogs of C2 (C2.01 (2-(2-hydroxy-4-methylbenzoyl)benzoic acid), C2.02 (2-(1-hydroxy-2-naphthoyl)benzoic acid), Fig 5C and 5D) and tested those in EMSAs as competitor for the La:fD1 interaction. As shown (Fig 5E and 5F compare to Fig 4A) C6.01 and C6.02 blocked the binding of La to fD1 at slightly lower concentrations than C6.00. Similar C2.01 and C2.02 impaired La:fD1 complex formation at lower concentration and both were not showing auto-fluorescence (Fig 5G and 5H compare to Fig 4E). Taken together, both 2D analogs of C2.00 and C6.00 blocked La:fD1 interactions in a similar range of concentrations.

### Analysis of the cell toxicity of 2D analogs of compound C2.00 and C6.00

**Fig 6:**
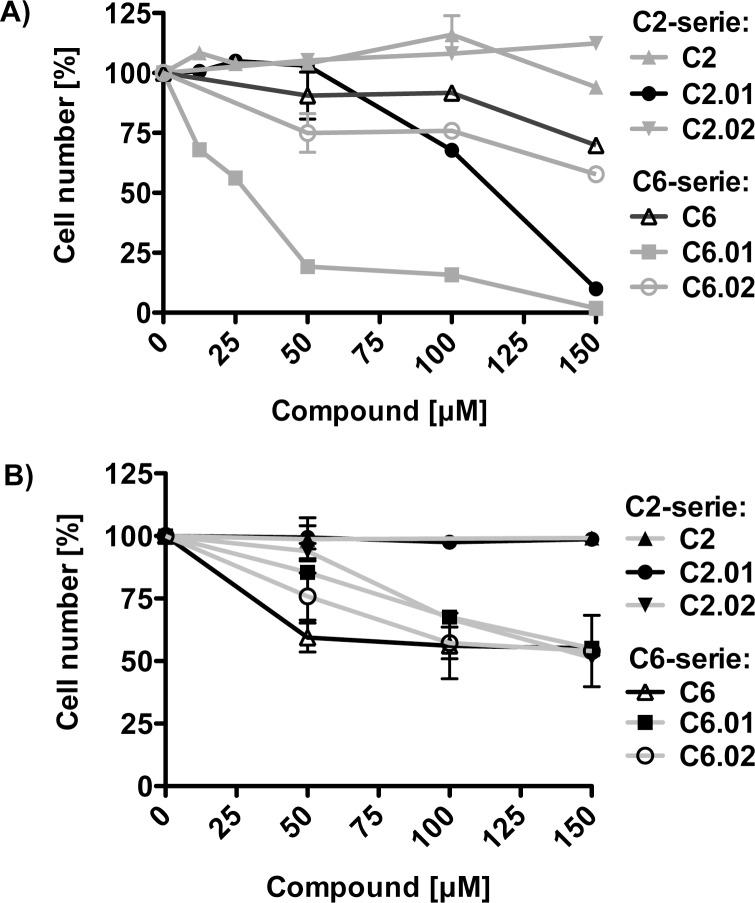
Cell toxicity of compound C2, C6, and their 2D analogs tested in a cancer cell line and normal fibroblasts. **A)** SCC 22B cells were treated with increasing concentrations of the indicated compound and 48 hours later cell numbers were determined (n = 3). **B)** MRC5 cells (fibroblast) were treated with increasing concentrations of the indicated compounds and 48 hours later cell numbers were determined using the CyQuant kit (n = 3).

After testing the compounds *in vitro* we wanted to assess all compounds (C2 2D-analogs: C2, C2.01, C2.02, and C6 2D analogs: C6, C6.01, C6.02) for their cell toxicity in cell culture experiments. For these initial studies, we used head and neck squamous cancer carcinoma cells (UM-SCC 22B (SCC 22B)[15], Fig 6A) and normal fibroblast cell line MRC5 (Fig 6B) and compared the cell toxicity of the six compounds. Cells were treated with compound concentrations of 12.5, 25, 50, 100, 150 μM and vehicle (DMSO) as control. At the endpoint of the experiment (after 48 hours), cell numbers were determined by applying the CyQuant assay. These experiments revealed that three compounds C6, C6.01, C6.02 reduced SCC 22B cell numbers even at low concentrations (Fig 6A, e.g. LaC6.01). The C2 and C2.02 had no effect on SCC 22B cells even at higher concentrations (Fig 6A), however, LaC2.01 was cytotoxic at concentrations higher than 50 μM.

To find out whether the compounds selectively affect cancer cells, we tested the cell toxicity of both compounds and their 2D analogs on normal fibroblasts (MRC5). C6, C6.01, C6.02, and C2.02 had a toxic effect on MRC5 cells, however, C2 and C2.01 had no toxic effect on those cell (Fig 6B). Most interestingly, compound C2.01 reduced the number of cancer cells, but did not display cell toxicity in normal fibroblasts.

### C2.01 impairs binding of La to specific cellular mRNAs

**Fig 7:**
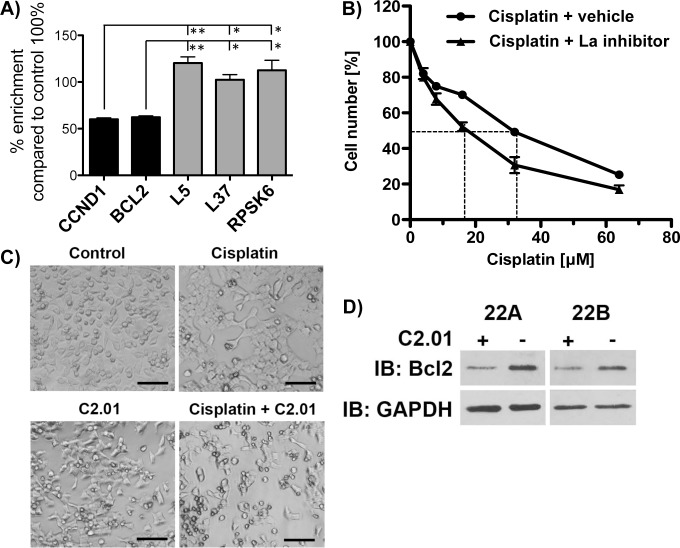
Compound C2.01 interferes with La:RNA binding in cells and sensitizes cells for cisplatin treatment. **A)** RNA Immunoprecipitation (RIP): RT-qPCR analysis of CCND1, Bcl2 and three different TOP mRNAs (L37, L5, RPSK6) associated with gfp-tagged La-WT in HEK293 cells treated with or without C2.01 (150 μM). The RNA binding between DMSO control and C2.01 treated cells was compared using the formula: [C2.01 treated (RIP/input) / DMSO control (RIP/input)] X 100 and the percentage of enrichment of mRNA in the control (DMSO treated) RIP pellet compared to the input material was set as 100%. **B)** SCC 22B cells were treated with increasing concentration of cisplatin and 50 μM C2.01 for 48 hours. Cell numbers were quantified using the CyQuant kit. **C)** Comparing phase contrast images of untreated SCC 22A cells, cells treated with cisplatin (at IC_50_ = 12 μM) or C2.01 (50 μM) alone, or in combination, demonstrates a dramatically reduced cell number in the co-treated sample. **D)** Immunoblot (IB) analysis for anti-apoptotic factor Bcl2 in cisplatin alone or cisplatin and C2.01 co-treated SCC 22A and SCC 22B cells. GAPDH serves as loading control.

Compound C2.01 acted as weak competitor in EMSAs but showed cytotoxicity in cancer cells and not in normal fibroblasts. Therefore, we asked whether C2.01 acts differently in cells compared to *in vitro* assays and performed RNA immunoprecipitation (RIP) experiments to test whether C2.01 inhibits binding of La to specific mRNAs in cells. For the RIP experiments, we used our established stably green fluorescence protein (gfp) or gfp-tagged La (_gfp_La) expressing HEK293 cell lines[29]. We found that cell proliferation and viability of HEK293 cells was impaired by C2.01 at concentration of 300 or 450 μM, but not at 150 μM or below (data not shown). The cells were control (DMSO)- and C2.01-treated (150 μM), and RIP experiments were performed using gfp-antibodies coupled to magnetic beads. RNA was prepared from input material as well as _gfp_La- and gfp-RIP pellets and analyzed by reverse transcription followed by quantitative PCR (RT-qPCR)[29]. We performed RT-qPCR for CCND1 and Bcl2, which are both bound efficiently via La-WT and La-RRM1+2(GS and TH unpublished data,[30]), and TOP mRNA also known to be bound by La-WT[54] but which are only weakly bound by La-RRM1+2 (Fig 2H). Strikingly, the compound C2.01 selectively impaired the binding of La to CCND1 and Bcl2 mRNA and had no effect on the binding of La to TOP mRNAs encoding ribosomal proteins RPL37, RPL5, and RPSK6 (Fig 7A). These data suggest that C2.01 can compete for the binding of La to specific mRNAs in cells but not *in vitro*.

### Compound C2.01 sensitizes cells for cisplatin treatment

We have recently shown that La protects head and neck cancer cell line SCC 22B against cisplatin-induced cell death by maintaining Bcl2 protein synthesis[15]. As presented above by RIP experiments, compound C2.01 impaired the binding of La to Bcl2 mRNA and, hence, we aimed to test whether compound C2.01 reduces Bcl2 protein level and sensitizes SCC 22B cells for cisplatin treatment.

Recently, we determined the cisplatin IC_50_ value for different head and neck cancer cell lines including SCC 22B[15]. As shown (Fig 6A), 50 μM of C2.01 was well tolerated for 48 hours by SCC 22B cells. To test whether C2.01 sensitizes SCC 22B cells for cisplatin, we first treated the cells with 50 μM C2.01 or vehicle (DMSO) for 4 hours. Subsequently, cisplatin at concentration of 4, 8, 16, 32 and 64 μM or vehicle was added. The cell number of cisplatin- or vehicle-treated cells were determined after 48 hours. These experiments showed that pretreatment of the cells with 50 μM of C2.01 sensitizes SCC 22B cells for cisplatin treatment as indicated by an IC_50_ shift from 32 to 18 μM (Fig 7B). Interestingly, C2.01 treatment sensitized SCC 22B cells to cisplatin-induced cell death, as recently shown for La depletion or overexpression of a domain negative La mutant[15]. The cytotoxic effect of C2.01, cisplatin or the combination of C2.01 with cisplatin on another head and neck cancer cell line, SCC 22A (UM-SCC 22A[14]), is also clearly visible by light microscopy (Fig 7C). The finding that C2.01 impaired the binding of La to Bcl2 mRNA and that C2.01 sensitizes cells for cisplatin might correlate with an impaired anti-apoptotic response to cisplatin. La is required to maintain Bcl2 protein synthesis in cisplatin-treated cells[15], suggesting that Bcl2 protein level are reduced in cells co-treated with C2.01 and cisplatin. Immunoblot analysis demonstrated that indeed Bcl2 protein levels are reduced in SCC 22B and SCC 22A cells treated with cisplatin and C2.01 (50 μM) when compared to vehicle-treated cells (Fig 7D).

In sum, we demonstrated for the first time that the compound C2.01 inhibits specific La:RNA interactions in cells and sensitizes cancer cells for cisplatin treatment, likley by reducing the binding of La to Bcl2 mRNA, leading to reduced expression of anti-apoptotic factor Bcl2.

## Discussion

Here we report the application of a high-throughput La:RNA fluorescence polarization assay (La-FP) that can be used to screen for compounds able to block the complex formation between the RNA-binding protein La and its target RNAs. Furthermore, we describe the initial characterization of identified compounds *in vitro* and in cells.

Since the La protein binds to a broad variety of RNAs via different combinations of RNA binding surfaces, the challenge of this project was to identify compounds able to block the binding of La to specific RNAs. We first compared the binding of La to three different substrates. All three substrates were bound by full length La (La wildtype, La-WT). Since the La motif is required for fPolyU binding, it was expected that fD1 but not fPolyU be bound via mutant La-RRM1+2. However, we found that the 5’end of TOP mRNA RPL5 was not strongly bound by La-RRM1+2. Our data indicate that binding of TOPf requires an additional binding surface in La, either the La motif or contacts in the C-terminal domain. We reported recently that the RNA chaperone activity of La requires amino acids in the C-terminus and that those residues are implicated in RNA binding has been previously suggested[30,39]. Hence, it might be possible that stable binding of TOP mRNAs requires amino acids located in the C-terminus of La. The finding that the binding of cap-TOPf was very similar to the binding of the TOPf and that an internal stretch of uridine nucleotides is important for binding, suggests that La is not directly binding the capped 5-terminus of TOP mRNA. More studies are required to define which domains of La are needed for a stable La:TOP mRNA interaction.

Based on these results, we postulate that a compound able to block the RRM2 binding surface would be a very good candidate, because La motif- and RRM1-mediated RNA binding (e.g. RNA polymerase III transcripts) would not be affected.

The initial hits identified in the La-FP assay had a promising IC_50_ value of 7.5 (C2) and 2.5 μM (C6). Validation in the orthogonal assays revealed that C2 was not very potent and blocked complex formation at 10 mM only. The analysis in orthogonal assays showed that the auto-fluorescence of C2 was probably leading to an aberrant fluorescence polarization IC_50_ value and that C2 only impaired La:RNA complex formation at 10 mM, highlighting the importance and effectiveness of EMSAs as an orthogonal assay. In contrast, C6 was validated as a potent inhibitor.

We observed that C6 was not a potent competitor for La:fPolyU compared to its action on La:fD1 or La:TOPf interactions, suggesting some selectivity; however, we have not yet demonstrated a strong difference in selectivity of C2 or C6 against different La:RNA complexes *in vitro*. Binding studies using 2D analogs C2.01, C2.02, C6.01, and C6.02 showed very similar effects on La:RNA intercations. Future work should focus on optimizing those hits into more potent and selective compounds.

Work by others and our recent work showed that La is posttranslational modified by phosphorylation and SUMOylation[29,30,54,56]. In RNA immunoprecipitation (RIP) experiments, phosphorylation as well as SUMOylation has been shown to modulate the association of La with mRNAs [29,54,56]. Hence, the finding that C2.01 impaired the association of La with specific cellular mRNAs, such as Bcl2 and CCND1, at μM concentration in cells suggests that C2.01 displays selectivity when tested in cell-based assays. It would be interesting to study whether C2 or C6 2D analog compounds would selectively compete for La:RNA interaction *in vitro* when phosphorylated and/or sumoylated recombinant La is used and whether they are also inhibiting the binding of other RNA-binding proteins to their target RNAs *in vitro*.

La is overexpressed in cancerous cells and promotes tumor-promoting and anti-apoptotic processes presumably by stimulating the translation of mRNAs under specific conditions such as cisplatin-treatment. To test cell toxicity of the compounds, we treated the SCC 22B cancer cell line and normal fibroblasts with the C2- and C6 2D analogs of compounds. Interestingly, we found that C2.01 and C6.01 preferentially reduced cancer cell numbers when compared to the effect on normal MRC5 cells. This important finding and the selective impairment of the interaction between La and Bcl2 and CCND1 mRNAs, suggests that the compounds might target La-supported cancer cell promoting processes [13,14,57]. We have recently shown that La protects against cisplatin-induced cell death and that La depletion or expression of a dominant negative La mutant sensitizes cells for cipslatin by reducing Bcl2 translation in cisplatin-treated cells[15]. Interestingly, we found that C2.01 not only sensitizes SCC 22B cells for cisplatin but also reduces Bcl2 expression. At this point, we do not know whether the less efficient binding of La to Bcl2 mRNA in C2.01-treated cells also correlates with less Bcl2 mRNA translation or whether other posttranscriptional mechanism accounting for reduced Bcl2 expression in cells co-treated with cisplatin- and C2.01. Future work should focus on evaluating whether C2.01 or improved molecules actually impair La-stimulated translation of specific mRNAs in cells e.g. treated with cisplatin.

Herein, we have revealed that small compounds can block La:RNA interactions *in vitro* and selectively in cells, however, at this point, the mode of action of the compounds is not identified and off-target effects might contribute to the cellular phenotype we observed. To identify potential off-target effects it will be crucial to test i) whether the compounds have an effect on La-depleted cells, ii) whether the compounds inhibit RRM-mediated RBP:RNA interactions only, or whether other RBP:RNA interactions mediated by other RNA-binding motifs are also affected, iii) whether compounds inhibit also other RBP:RNA interactions by performing RIP experiments for other RBPs, iv) whether global translation is impaired, v) and it should be tested whether biochemical processes leading to hepatoxicity are impaired and whether cytochrome P450 activity is reduced. In addition those molecules might be applicable and useful as molecular tool to disrupt La:RNA interactions in *in vitro* assays such as *in vitro* translation, RNA chaperone assays, and RNA-binding assays.

In summary, we have established for the first time a robust high-throughput assay applicable to identify small compounds inhibiting La:RNA interactions *in vitro*. We have shown that C2.01 impairs binding of La to specific mRNAs in cells and thereby mimics the effect of La depletion in head and neck cancer cells by reducing Bcl2 protein expression and sensitization to cisplatin.

## Supporting information

S1 Table Oligonucleotides used in this study. (PDF)

S1 Fig Predicted secondary structures of RNA oligoribonucleotides used in this study. Secondary structures were predicted using the mfold web server at http://unafold.rna.albany.edu.
(PDF)
