# Supplementary material for: Applying a high-throughput fluorescence polarization assay for the discovery of chemical probes blocking La:RNA interactions in vitro and in cells
Source: PLoS One. 2017 Mar 14;12(3):e0173246. doi: 10.1371/journal.pone.0173246 (PMC5349447; doi:10.1371/journal.pone.0173246)
Supplement: S1 Table — (PDF) [file pone.0173246.s001.pdf]

Oligonucleotides:

Unlabeled and fluorescence labeled RNA oligonucleotides were synthesized by Integrated DNA Technologies, Inc.:

fD1: 5′-FAM-rGrCrUrGrCrCrCrArGrGrArArGrArGrCrCrCrCrArGrCrCrArUrGrGrArArCrArCrCrArG rCrUrCrCrUrGrUrGrCrUrGrC-3′

fPolyU: /56-FAMN/rUrGrCrUrGrUrUrUrU

TOPf: rCrCrCrUrUrUrUrCrCrCrArCrCrCrCrCrUrArGrCrGrCrCrGrCrUrGrGrGrCrCrU/36-FAM/

TOP-WT: rCrCrCrUrUrUrUrCrCrCrArCrCrCrCrCrUrArGrCrGrCrCrGrCrUrGrGrGrCrCrU

TOP-mu1: rCrCrCr**<u>A</u>**r**<u>A</u>**r**<u>A</u>**r**<u>A</u>**rCrCrCrArCrCrCrCrCrUrArGrCrGrCrCrGrCrUrGrGrGrCrCrU.

Mutation indicated in bold and underlines.

TOP-mu2: r**<u>G</u>**r**<u>G</u>**rCrCrCrUrUrUrUrCrCrCrArCrCrCrCrCrUrArGrCrGrCrCrGrCrUrGrGrGrCrCrU

Mutation indicated in bold and underlines.

The capped and fluorescence labeled oligoribonucleotide was ordered by TriLink BioTechnologies, Inc.:

CapTOPf: 5'-(N7MeGppp)rCrCrCrUrUrUrUrCrCrCrArCrCrCrCrCrUrArGrCrGrCrCrGrCrUrGrGrGrCr CrU (FAM) -3'

Sommer et al. S1 Table
