# Supplementary material for: Applying a high-throughput fluorescence polarization assay for the discovery of chemical probes blocking La:RNA interactions in vitro and in cells
Source: PLoS One. 2017 Mar 14;12(3):e0173246. doi: 10.1371/journal.pone.0173246 (PMC5349447; doi:10.1371/journal.pone.0173246)
Supplement: S1 Fig — Secondary structures were predicted using the mfold web server at http://unafold.rna.albany.edu. (PDF) [file pone.0173246.s002.pdf]

A)

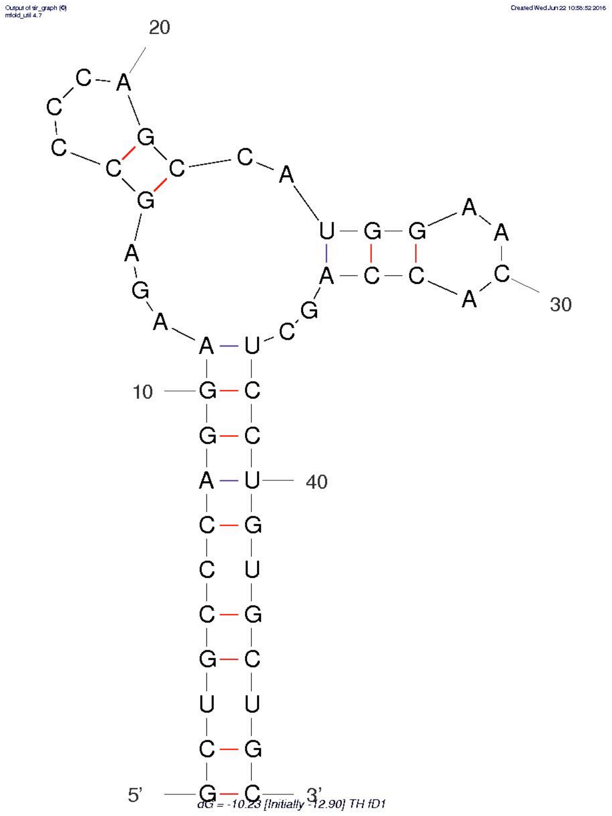

B)

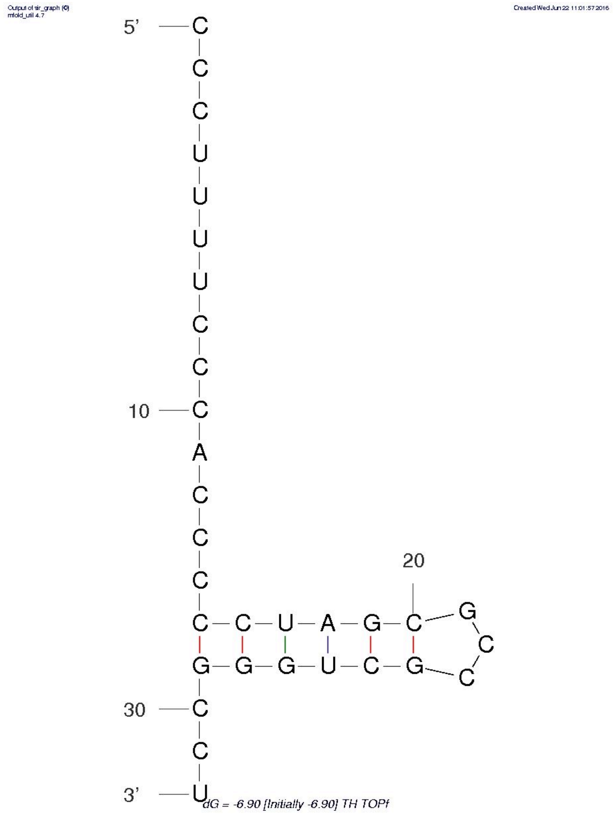

CCND1 mRNA derived oligoribonucleotide: D1
5’-GCUGCCCAGGAAGAGCCCCAGCCAUGGAACACCAGCUCCUGUGCUGC
- Only predicted structure (mfold)
- ΔG = -10.23

RpL5 mRNA derived oligoribonucleotide: TOP
5’-CCCUUUUCCCACCCCCUAGCGCCGCUGGGCCU
- Only predicted structure (mfold)
- ΔG = -6.90

C) No structure predicted for model oligoribonucleotde PolyU:
5’-UGCUGUUUU

Sommer et al. S1 Fig
